# Childhood Obesity and Its Correlation With Vitamin D: A Systematic Review

**DOI:** 10.7759/cureus.85047

**Published:** 2025-05-29

**Authors:** America Aldana, Welaa Aljaroudi, Cheryl Estes, Fatima Rizvi, Chinwe Okeke-Moffatt, Viktoria Minakova

**Affiliations:** 1 Biochemistry and Genetics, Saint James School of Medicine, Arnos Vale, VCT

**Keywords:** childhood obesity, fasting glucose, lipids, pth, vitamin d deficiency

## Abstract

Several studies have identified a correlation between vitamin D deficiency and childhood obesity. The aim of this study is to perform a systematic review of data collected to determine how significant the effect of vitamin D deficiency is on childhood obesity and to determine the extent to which vitamin D deficiency has a predictive association with health status in the pediatric population, where obesity is a concern. This review will add value to the current body of knowledge and provide valuable clinical guidance in avoiding the negative effects of vitamin D deficiency on childhood obesity.

A systematic literature review was conducted through a variety of resources, including MEDLINE, Google Scholar, PubMed, and JSTOR. The following search terms were used to identify relevant studies discussing the correlation between childhood obesity and vitamin D deficiency: "vitamin D", "childhood obesity", "pediatrics", and "vitamin D deficiency". Citations were screened and assessed for quality via Rayyan (Rayyan Systems Inc., Cambridge, MA), a web application. If an article met the exclusion criteria, it was excluded from the analysis. Of the selected studies, data on the association between vitamin D deficiency and childhood obesity were presented and discussed.

The majority of the chosen investigative studies found a statistically significant association between vitamin D deficiency and childhood obesity. The studies reported a significant inverse correlation between body mass index (BMI) and vitamin D levels, and the pooled correlation coefficient (r) was approximately -0.299 (p<0.01), indicating a moderate inverse relationship. Additionally, the majority of the studies stated that the chances of having vitamin D deficiency increase with a pooled odds ratio of 2.6 (95% confidence interval (CI): 1.4-4.76). These findings also suggested potential bidirectional relationships. Obesity may reduce vitamin D levels (due to sequestration of vitamin D in fat tissue), and low vitamin D levels might also influence body weight and fat accumulation through effects on metabolism and insulin sensitivity. Clarifying the direction and nature of this relationship is important for treatment strategies, screening obese children for vitamin D deficiency, or vice versa, improving patient care. Understanding this relationship could help in developing targeted nutritional or physical activity guidelines, supplementation programs, or obesity prevention campaigns.

Along with obesity, vitamin D further demonstrates relationships in more specific metabolic disorders such as impaired fasting glucose, lipids, and parathyroid hormone (PTH), although with some conflicting findings. The mechanism behind these associations implicating vitamin D as a causal factor remains overall unclear. However, evidence certainly supports a close relationship in these metabolic concerns, to support the potential coexistence of these concerns and to consider broad testing and treatment accordingly.

## Introduction and background

Obesity continues to be a serious health issue in the United States among children and adolescents ranging from two to 19 years of age [1]. Characterized as excess body fat, obesity is commonly measured using body mass index (BMI), a calculation of weight in pounds divided by height in inches squared, multiplied by 703 [2]. According to the Centers for Disease Control and Prevention (CDC), a BMI greater than or equal to the 95th percentile on the BMI-for-age growth chart is considered to be symptomatic of various health problems [2]. There is also a general consensus on a global trend of the rapid growth of obesity rates, which contributes to health ailments [3]. Research indicates that overweight youth suffer from chronic conditions such as insulin resistance, inflammation, and hypertension [4].

Vitamin D, also known as calciferol, is a fat-soluble vitamin that can be found in significant concentrations in foods such as oily fish. Lower concentrations of vitamin D can be found in egg yolks, red meat, and beef liver. Another source of vitamin D, which is of great importance, is natural sunlight. Vitamin D acquired from meals and the sun is physiologically inactive and requires two hydroxylation reactions in the body to become active. The liver is the site of the initial hydroxylation where vitamin D is converted to 25-hydroxyvitamin D (25(OH)D), commonly known as "calcidiol". The second hydroxylation occurs in the kidneys and results in the physiologically active 1,25-dihydroxyvitamin D (1,25(OH)2D), also known as "calcitriol". While vitamin D is obtained from dietary sources and sun exposure, levels may be inadequate and are further impaired by environmental, cultural, and genetic factors [3].

A deficiency in vitamin D occurs when daily intake is lower than required. Some factors include cultural clothing that, traditionally, covers most of the body [2]. Limited sun exposure prevents the kidneys from converting 25(OH)D to its active form, and vitamin D absorption from the digestive system becomes insufficient [5]. According to the Office of Dietary Supplements, vitamin D deficiency is defined as a level below 30 nmol/L, which may be linked to a negative health status. Some people are potentially at risk of hypovitaminosis D at 30-50 nmol/L (12-20 ng/mL). Levels of 50 nmol/L (20 ng/mL) or greater are sufficient for most people. In contrast, a serum 25(OH)D concentration of more than 75 nmol/L (30 ng/mL) is necessary to maximize the effect of vitamin D on calcium, bone, and muscle metabolism [6]. The serum 25-hydroxyvitamin D concentrations and their impact on health, as summarized by the National Institutes of Health, are presented in Table 1 [6].

**Table 1:** Serum 25(OH)D concentrations and their impact on health *Serum concentrations of 25(OH)D are reported in both nmol/L and ng/mL. One nmol/L = 0.4 ng/mL, and 1 ng/mL = 2.5 nmol/mL. 25(OH)D: 25-hydroxyvitamin D, nmol/L: nanomoles per liter, ng/mL: nanograms per milliliter Source: [6]

nmol/L*	ng/mL*	Health status
<30	<12	Associated with vitamin D deficiency, which can lead to rickets in infants and children and osteomalacia in adults
30 to <50	12 to <20	Generally considered inadequate for bone and overall health in healthy individuals
≥50	≥20	Generally considered adequate for bone and overall health in healthy individuals
>125	>50	Linked to potential adverse effects, particularly at >150 nmol/L (>60 ng/mL)

Obesity is a multifactorial condition influenced by a combination of genetic, dietary, and lifestyle factors [7]. Research suggests that obesity may reduce circulating vitamin D levels due to the sequestration of vitamin D in fat tissue [2]. Additionally, low vitamin D levels influence body weight and fat accumulation through their effects on metabolism and insulin sensitivity [2]. While vitamin D regulates metabolic processes that have positive long-term health effects, such as enhancing fat metabolism and insulin sensitivity, supplementing vitamin D alone may not reverse obesity, since it is a multifactorial condition [7]. It is crucial to understand the relationship between obesity and vitamin D, since obesity is a chronic condition that affects various diseases, and vitamin D is an important metabolic regulator [3,4,8-10]. Understanding this relationship is important for early diagnosis and intervention, enabling better treatment and prevention of long-term health complications.

Vitamin D has strong documentation as a "global health issue", with multiple studies providing evidence linking deficiency to chronic disorders such as cardiovascular disease and autoimmune disorders [3,4,8-10]. However, despite what should be conclusive evidence, disagreements remain as to the strength of the correlation, exactly how to define the deficiency, and treatment [8]. The importance of vitamin D to functional health, such as in bones and development, seems quite clear, but the extent and direct association to other health effects are still inconclusive, especially in the pediatric population [10,11]. With the extent of controversial and inconclusive research regarding vitamin D, we want to review the recent literature on vitamin D and childhood obesity to investigate any trends of association, or lack thereof, in the health status of obese pediatric subjects.

This article was previously presented as a poster at the Saint James School of Medicine (SJSM) Science Day Meeting on December 6, 2021.

## Review

Methods

**Figure 1:**
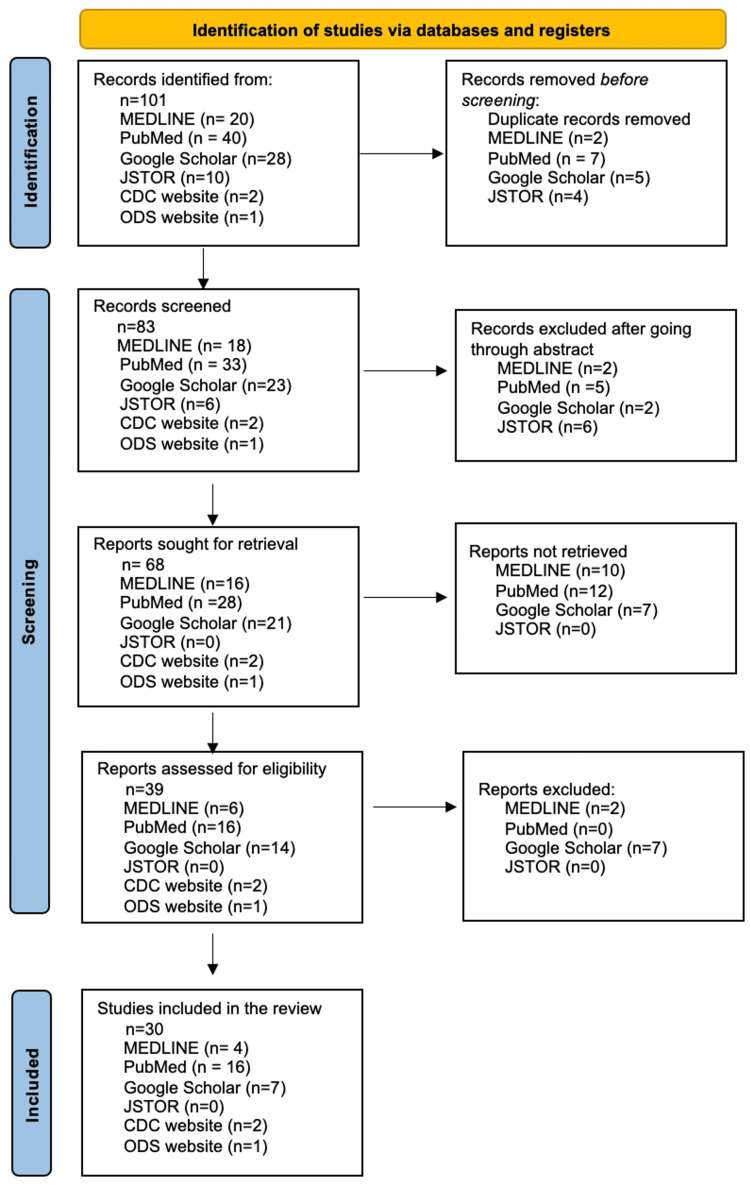
Flowchart for screening and identifying eligible studies CDC: Centers for Disease Control and Prevention, ODS: Office of Dietary Supplements

Research methods included a comprehensive systematic literature review conducted through a variety of resources, including MEDLINE, Google Scholar, PubMed, and JSTOR. The following search terms were used to identify relevant studies discussing the correlation between childhood obesity and vitamin D deficiency: "vitamin D", "childhood obesity", "pediatrics", and "vitamin D deficiency". Citations were screened and assessed for quality via Rayyan (Rayyan Systems Inc., Cambridge, MA), a web application. If an article met the exclusion criteria, it was excluded from the analysis. Two review authors assessed study eligibility, and risk of bias was assessed, with a third reviewer resolving any discrepancies. Data screening and extraction were completed independently by each reviewer. A flowchart for screening and identifying eligible studies is presented in Figure 1.

The inclusion criteria for this research were as follows: scholarly or peer-reviewed source, relevant article within the last 15 years (the cutoff was 2006), and prospective cohort studies, cross-sectional studies, randomized clinical trials, and meta-analysis articles published in the English language only. The articles chosen were those that examine the possible association between childhood obesity and vitamin D deficiency. Articles that were excluded were as follows: publications potentially used for marketing purposes, articles in foreign languages, articles dated prior to 2006, literature reviews, and duplicate articles.

Outcome measures included anthropometric variables (BMI, weight, and height), fasting concentrations of serum 25-hydroxyvitamin D (25(OH)D), parathyroid hormone (PTH), calcium and phosphate, mean lipid levels, fasting blood sugar, and insulin sensitivity.

Results

In this systematic review, we focus on investigating the relationship between vitamin D levels and obesity in the pediatric population, as presented in Table 2.

**Table 2:** Association between childhood obesity and vitamin D deficiency 25(OH)D: 25-hydroxyvitamin D, BMI: body mass index, CI: confidence interval, SDS: standard deviation score, PR: prevalence ratio, OR: odds ratio

Authors (year)	Design	Mean sample age	Findings
Ekbom and Marcus (2016) [12]	Prospective cross-sectional study, N=202	11.2 years	33.2% of the obese Swedish children were vitamin D deficient (25 (OH)D < 30 nmol/L). No significant relationship was observed between vitamin D deficiency and BMI (p=0.7).
Kumaratne et al. (2017) [13]	Retrospective study, N=234	16 years	Vitamin D deficiency (23.1±6.6 ng/mL) was significantly associated with overweight or obesity in Hispanic American adolescents. A BMI of ≥85 kg/m^2 ^is a good indicator of vitamin D deficiency with OR of 2.02, CI of 1.11-3.69, X^2^=5.37, and p=0.021.
Çizmecioğlu et al. (2008) [14]	Cross-sectional study, N=301	14.2±1.8 years	In overweight and obese children, a negative correlation between BMI and vitamin D level was observed (r=-0.186, p<0.01) (vitamin D deficiency < 10 ng/mL).
Wakayo et al. (2016) [15]	Cross-sectional study, N=174	14.5 years	A negative correlation between vitamin D level and BMI in Ethiopian school children (r=-0.233, p=0.002) was observed. Vitamin D deficiency was defined as <50 nmol/L.
Bilici et al. (2019) [16]	Cross-sectional study, N=96	13.1±2.4 years	At the beginning of the study, 54 (56.2%) obese adolescents had vitamin D deficiency (8.1±3.3 ng/mL) compared to 42 (43.8%) obese adolescents who were vitamin D sufficient (20.7±6.2 ng/mL). Treatment with vitamin D supplementation of 2,000 IU/day was initiated for three months for the vitamin D-deficient group. Vitamin D level increased after the three-month treatment to 23.49±9.7 ng/mL compared to 7.58±2.7 ng/mL before treatment. Additionally, BMI SDS was significantly reduced with vitamin D supplementation (p=0.008). Only 23 (54.7%) were assessed at the end of the study; 12 (22.2%) subjects declined treatment, four (9.5%) subjects stopped treatment, treatment was not taken daily by nine (21.4%) subjects, and six (14.2%) subjects did not complete the three-month visit.
Chen et al. (2021) [17]	Cross-sectional study, N=1,510	44±8.2 months	Obese children had lower levels of serum 25(OH)D (25.51±7.34 ng/mL, p=0.035) when compared to non-obese children (28.07±7.28 ng/mL). The association between vitamin D deficiency and obesity (5.23%) was much higher than vitamin D sufficiency (2.02%) and insufficiency (2.14%) (p=0.043). BMI was not significantly different between obese and non-obese children with or without vitamin D deficiency (p>0.05).
Lenders et al. (2009) [18]	Cross-sectional study, N=58	14.9±1.4 years	17 obese subjects, with mean BMI of 36±5 kg/m^2^, were 25(OH)D deficient (25(OH)D < 20 ng/mL). An inverse relationship was observed between the body fat mass and 25(OH)D level (p<0.05). As the percentage of body fat mass increases by 1%, the chances of having vitamin D deficiency increase by 1.78 (after adjusting for the core variables) (adjusted OR: 1.78, 95% CI: 1.27-2.50, p=0.001).
Durá-Travé et al. (2017) [19]	Cross-sectional study, N=546	3.2-15.8 years	Results indicate that hypovitaminosis D is associated with BMI. Obese (68.2%) and severely obese (81.1%) subjects had the highest level of hypovitaminosis D (26.18±7.0 ng/mL and 23.09±8.24 ng/mL, respectively) compared to normal (58.1%) and overweight (55%) subjects (p=0.001). Z-score based on BMI: normal, -1.0 to +1.0 (15th percentile to 85th percentile); overweight, more than 1.0 (85th percentile); obese, more than 2 (97th percentile); and severely obese, more than 3 (99th percentile).
Esmaili et al. (2020) [20]	Cross-sectional study, N=2,594	12.2 years	Vitamin D deficiency was more observed in metabolically unhealthy obese (85.3%) when compared to other metabolic phenotypes of obesity (metabolically healthy obese, metabolically non-healthy non-obese, and metabolically healthy non-obese) (p<0.001).
Guo et al. (2021) [21]	Cross-sectional study, N=414	1 year	In one-year-old Chinese infants, an association was observed between vitamin D deficiency and infant obesity (adjusted OR: 2.74, 95% CI: 1.20-6.25, with 25(OH)D ≥ 75 nmol/L as a reference for sufficient vitamin D level). Also, results suggest an inverse linear relation between 25(OH)D level and BMI (β=-0.017, p=0.004).
Pereira-Santos et al. (2015) [22]	Systematic review and meta-analysis, articles included: 23	Different age groups	Compared to the eutrophic group, obese individuals were 35% more vitamin D deficient (PR: 1.35, 95% CI: 1.21-150), regardless of age.
Fiamenghi and Mello (2021) [23]	Systematic review and meta-analysis, articles included: 20, N=24,600	9 years	In obese children and adolescents, low vitamin D level was associated with obesity (relative risk: 1.41) (95% CI: 1.26-1.59) (I^2^=89%, p<0.01) (obesity BMI ≥ 95, vitamin D deficiency < 20 ng/mL).
Reymann et al. (2014) [9]	Cross-sectional study, n=64 (obese), n=32 (healthy)	11 years	Vitamin D deficiency was more observed in obese children (56%) when compared to control healthy children (16%) (p=0.0001). Vitamin D deficiency is defined as a level less than or equal to 37.5 nmol/L (15 ng/mL), insufficiency as between 37.5 nmol/L and 50 nmol/L (15-20 ng/mL), and sufficiency as a level greater than 50 nmol/L.
Denova-Gutiérrez et al. (2019) [24]	Cross-sectional study, N=533	11.6 years	An inverse relationship was found between vitamin D levels and being overweight or obese (90%, p<0.001). In other words, vitamin D insufficiency and deficiency were more common among overweight and obese children.
Yahyaoui et al. (2019) [25]	Case-control study, N=60 (30 obese and 30 control non-obese)	Obese: 104.43±28.65 months, control: 106.13±30.68 months	Tunisian obese children had higher levels (94%) of vitamin D deficiency (median vitamin D level: 17.2±5.9 ng/mL) compared to control non-obese children (80%) (p=0.002). A negative correlation was observed between vitamin D level and BMI (p=0.001, r=-0.51).
Olson et al. (2012) [8]	Case-control study, n=414 (obese), n=89 (non-obese)	11 years	Across different ethnic groups in North Texas, vitamin D deficiency (<20 ng/mL) was observed in obese children when compared to the control (OR: 4.0, 95% CI: 2.0-6.6).

Moreover, some research papers suggested a relationship between low vitamin D levels and insulin resistance. Based on that, we explored and presented this association in obese children in Table 3.

**Table 3:** Vitamin D deficiency and insulin resistance in obese children 25(OH)D: 25-hydroxyvitamin D, QUICKI: Quantitative Insulin Sensitivity Check Index, HOMA-IR: Homeostatic Model Assessment for Insulin Resistance, BMI: body mass index, OR: odds ratio, CI: confidence interval, type 2 DM: type 2 diabetes mellitus

Authors (year)	Design	Mean sample age	Findings
Ekbom and Marcus (2016) [12]	Prospective cross-sectional study, N=202	11.2 years	Obese Swedish children with vitamin D deficiency had an increased risk of developing impaired fasting glycemia (9.1%) (p=0.01). Also, results indicate that there was an inverse relationship between 25(OH)D and fasting glucose (r=-0.15, p<0.05) and fasting insulin (r=-0.15, p<0.05).
Bilici et al. (2019) [16]	Cross-sectional study, N=96	13.17±4.2 years	At the beginning of the study, 54 (56.2%) obese adolescents had vitamin D deficiency (8.1±3.3 ng/mL) compared to 42 (43.8%) obese adolescents who were vitamin D sufficient (20.7±6.2 ng/mL). Treatment with vitamin D supplementation of 2,000 IU/day was initiated for three months for the vitamin D-deficient group. When comparing the values of fasting glucose (p=0.81) and fasting insulin (p=0.27) before and after receiving the three-month vitamin D supplementation, no significant difference was observed. Only 23 (54.7%) were assessed at the end of the study; 12 (22.2%) subjects declined treatment, four (9.5%) subjects stopped treatment, treatment was not taken daily in nine (21.4%) subjects, and six (14.2%) subjects did not complete the three-month visit.
Lenders et al. (2009) [18]	Cross-sectional study, N=58	14.9±1.4 years	In obese subjects, no relationship was observed between vitamin D deficiency and insulin index (p>0.05).
Fiamenghi and Mello (2021) [23]	Systematic review and meta-analysis, articles included: 20, N=24,600	9 years	In children and adolescents, low vitamin D level was associated with obesity and insulin resistance (p<0.001) (obesity BMI > 95, vitamin D deficiency < 20 ng/mL).
Reymann et al. (2014) [9]	Cross-sectional study, n=64 (obese), n=32 (healthy)	11 years	Vitamin D deficiency was observed more in obese children (56%) when compared to healthy control children (16%). By using a multiple linear regression model, obese children with vitamin D deficiency had lower insulin sensitivity when compared to obese and normal children with vitamin D insufficiency (low QUICKI and HOMA-IR, p=0.045). Also, a significant relationship was observed between QUICKI and BMI and age (p<0.001). Vitamin D deficiency is defined as a level less than or equal to 37.5 nmol/L (15 ng/mL), insufficiency as between 37.5 nmol/L and 50 nmol/L (15-20 ng/mL), and sufficiency as a level greater than 50 nmol/L.
Denova-Gutiérrez et al. (2019) [24]	Cross-sectional, N=533	11.6 years	There was an inverse relationship between vitamin D and overweight and obesity (90%, p<0.01). Moreover, a higher odds of insulin resistance was observed with low level of serum vitamin D level (32.8%) (OR: 2.9, 95% CI: 1.1-7.2, p-trend: 0.03) (BMI: normal, 367±68.9% kg/m^2^; overweight, 118±22.1% kg/m^2^; obese, 49±9.0% kg/m^2^).
Olson et al. (2012) [8]	Case-control study, n=414 (obese), n=89 (non-obese)	11 years	Across different ethnic groups in North Texas, vitamin D deficiency (below 20 ng/mL) was observed in obese children when compared to controls (OR: 4.0, 95% CI: 2.0-6.6). A negative correlation was observed between vitamin D level and HOMA-IR (r=-0.19, p=0.001) and two-hour glucose test (r=-0.12, p=0.04). Vitamin D deficiency is considered a risk factor for type 2 DM.

Additionally, we examined lipid metabolism in obese children with vitamin D deficiency, as presented in Table 4.

**Table 4:** Vitamin D deficiency and lipid metabolism in obese children LDL: low-density lipoprotein

Authors (year)	Design	Mean sample age	Findings
Ekbom and Marcus (2016) [12]	Prospective cross-sectional study, N=202	11.2 years	A significant relationship was observed between cholesterol (p=0.05), triglyceride (p=0.003), and vitamin D deficiency in obese Swedish children; higher lipid values were associated with vitamin D deficiency.
Kumaratne et al. (2017) [13]	Retrospective study, N=234	16 years	When compared to subjects with normal level of vitamin D, overweight and obese Hispanic American adolescents with vitamin D deficiency had significantly higher cholesterol (165±28.6 mg/dL versus 145.7±27.5 mg/dL, p=0.003), LDL (92.7±25.7 mg/dL versus 80.8±21.4 mg/dL, p=0.007), and triglycerides (148.9±97.1 mg/dL versus 90.6±40.7 mg/dL, p=0.000).
Bilici et al. (2019) [16]	Cross-sectional study, N=96*	13.17±4.2 year	At the beginning of the study, 54 (56.2%) obese adolescents had vitamin D deficiency (8.1±3.3 ng/mL) compared to 42 (43.8%) obese adolescents who were vitamin D sufficient (20.7±6.2 ng/mL). Treatment with vitamin D supplementation of 2,000 IU/day was initiated for three months for the vitamin D-deficient group. Only total cholesterol (169.7±31.9 mg/dL versus 157.7±28.6 mg/dL, p=0.018) and LDL (103.6±30.1 mg/dL versus 93.9±29 mg/dL, p=0.004) were significantly reduced; however, no change in triglyceride level was observed (120.3±49.2 mg/dL versus 114.5±64.5 mg/dL, p=0.2). (*Only 23 (54.7%) were assessed at the end of the study; 12 (22.2%) subjects declined treatment, four (9.5%) subjects stopped treatment, treatment was not taken daily in in nine (21.4%) subjects, and six (14.2%) subjects did not complete the three-month visit.)

Lastly, parathyroid hormone, calcium, and phosphate levels were examined in obese children with vitamin D deficiency, as presented in Table 5.

**Table 5:** Vitamin D deficiency in obese children and PTH, calcium, and phosphate levels PTH: parathyroid hormone, 25(OH)D: 25-hydroxyvitamin D, BMI: body mass index

Authors (year)	Design	Mean sample age	Findings
Çizmecioğlu et al. (2008) [14]	Cross-sectional study, N=301	14.2±1.8 years	In overweight and obese children, no association was observed between vitamin D level and intact PTH (r=-0.074, p=0.202).
Bilici et al. (2019) [16]	Cross-sectional study, N=96*	13.17±4.2 years	At the beginning of the study, 54 (56.2%) obese adolescents had vitamin D deficiency (8.1±3.3 ng/mL) compared to 42 (43.8%) obese adolescents who were vitamin D sufficient (20.7±6.2 ng/mL). Treatment with vitamin D supplementation of 2,000 IU/day was initiated for three months for the vitamin D-deficient group. PTH was significantly reduced (before: 69.3±22.0 mg/mL, after: 56.3±18.4 mg/mL, p=0.016), while there was no effect of vitamin D supplementation on calcium (before: 9.7±0.30 mg/dL, after: 9.73±0.31 mg/dL, p=0.31) or phosphorus levels (before: 4.5±0.76 mg/dL, after: 4.37±0.65 mg/dL, p=0.37). (*Only 23 (54.7%) were assessed at the end of the study; 12 (22.2%) subjects declined treatment, four (9.5%) subjects stopped treatment, treatment was not taken daily in in nine (21.4%) subjects, and six (14.2%) subjects did not complete the three-month visit.)
Lenders et al. (2009) [18]	Cross-sectional study, N=58	14.9±1.4 years	17 obese subjects were 25(OH)D-deficient (25(OH)D < 20 ng/mL); however, PTH was within the normal level.
Durá-Travé et al. (2017) [19]	Cross-sectional study, N=546	9.5 years	Results indicate that PTH is positively correlated with BMI (p<0.01, r=0.268). The frequency of high levels of hyperparathyroidism was observed more in obese (9.1%) and severely obese (26.1%) subjects compared to normal (2.5%) and overweight (6.1%) subjects (p=0.001). No association was found between BMI and calcium and phosphate. Z-score based on BMI: normal, -1.0 to +1.0 (15th percentile to 85th percentile); overweight, more than 1.0 (85th percentile); obese, more than 2 (97th percentile); and severely obese, more than 3 (99th percentile).

Discussion

Pediatric Obesity and Vitamin D Deficiency

Obesity and vitamin D: Based on our findings, a general summary can be stated that evidence appears to support a link between the relative risk of vitamin D deficiency and obesity [23]. Many of the studies also highlighted findings that obesity was not isolated to vitamin D, but other issues, such as high triglycerides [13] and bone alterations [26], are often concurrently associated concerns.

Serum concentrations of 25-hydroxyvitamin D (25(OH)D) are used to determine vitamin D levels. Vitamin D deficiency is defined as less than 12 ng/mL, insufficiency as between 12 and 20 ng/mL, and sufficiency as equal to or greater than 20 ng/mL [5,6].

Both pediatric obesity and vitamin D insufficiency have been classified as epidemics in the United States. Obesity is prevalent among children aged 6-11 years (18%) and adolescents aged 12-19 years (18.4%) according to the National Health and Nutrition Examination Survey (NHANES) 2009-2010 survey. Similarly, statistics from the NHANES 2003-2006 show that roughly 21% of normal-weight kids are vitamin D deficient, whereas the prevalence of vitamin D deficiency in obese children grows with excess adiposity, reaching 49% in the severely obese [2].

Vitamin D and adiposity: Obesity increases the risk of vitamin D insufficiency. Vitamin D deficiency is hypothesized to result from the vitamin's preference for deposition in body fat compartments, leaving it unavailable for conversion to 25(OH)D. Early studies show that obese people are only about half as effective at converting vitamin D to 25(OH)D, whether taken orally or by cutaneous synthesis after UVB exposure [2]. Human experiments show that adipose tissue is a primary depositor of vitamin D and its metabolites. Some experts disagree with this theory, purporting that when concentrations are based on BMI, vitamin D bioavailability does not differ between normal-weight and obese individuals, but rather, the difference is due to a dilutional effect of circulating 25(OH)D concentration [2].

Lipids: While there was not an extensive amount of research identified, it is worth noting the discovered relationship between vitamin D levels and dyslipidemia. Supportive findings indicate the relationship may be low-density lipoprotein (LDL)-specific and not as clearly associated with triglycerides; however, with the deleterious impact of high LDL on cardiovascular health, this finding should not go unrecognized [16]. This finding could potentially have ethnic and geographic considerations, in particular as shown in a study of Hispanic children and Swedish children [12,13]. A positive association between lipid levels and vitamin D has raised concerns for proper laboratory screening and identification. This should raise potential concerns in the area of lifestyle as an underlying exacerbating factor of these associations.

A particular study yields an interesting conversation on the potential molecular mechanisms behind the link between vitamin D and lipids. The root of this relationship appears to lie within the peroxisome proliferator-activated receptor gamma (PPARγ) receptor, an established regulator of adipocyte maturation. There is growing evidence on the interaction of vitamin D as an inhibitor of PPARγ, thus, in effect, demonstrating the relationship of vitamin D and adiposity on a molecular level [8].

Vitamin D, calcium, and PTH: The functions of vitamin D and PTH in obesity are heavily debated. Vitamin D and PTH are well known for their significance in calcium balance and bone metabolism. Obesity is linked to the vitamin D endocrine system [9]. Obesity has been linked to decreased serum levels of 25-hydroxyvitamin D and increased serum levels of PTH. Vitamin D deficiency has been linked to an increase in BMI. PTH has been proposed as a stand-alone predictor of obesity. PTH stimulates the renal hydroxylation of 25-hydroxyvitamin D to its active form, 1,25-dihydroxyvitamin D, which increases calcium influx into adipocytes. Intracellular calcium increases lipogenesis by activating fatty acid synthase and inhibits lipolysis by activating phosphodiesterase 3B, which lowers catecholamine-induced lipolysis in these cells. Both of these effects would induce fat tissue to accumulate lipids [9]. A particular study suggested that PTH is positively correlated with BMI (p<0.01, r=0.268). The frequency of high levels of hyperparathyroidism was observed to be higher in obese (9.1%) and severely obese (26.1%) subjects compared to normal (2.5%) and overweight (6.1%) subjects (p=0.001) [19]. However, no association was found between BMI and calcium and phosphate.

Fasting blood sugar: Investigation into metabolic impairments associated with vitamin D levels yielded potentially significant findings. Several studies were able to demonstrate an association between fasting glucose, insulin resistance, and general diabetes or at most metabolic syndrome [9,27]. Several studies reported statistically significant results for vitamin D deficiency and insulin resistance either by linear regression or odds ratio [9,23]. Low vitamin D levels were linked to obesity and insulin resistance in children and adolescents (p<0.001) [23,27]. When comparing obese children to healthy children, it was discovered that vitamin D deficiency was more common in obese children (56%) compared to healthy control children (16%). Also, obese children with vitamin D deficiency exhibited reduced insulin sensitivity (low Quantitative Insulin Sensitivity Check Index (QUICKI) and Homeostatic Model Assessment for Insulin Resistance (HOMA-IR), p=0.045). In addition, there was a significant connection between QUICKI and BMI and age (p<0.001) [9].

This association extends further from what would be considered obesity-related causes. Of interest, a study conducted in Finland [28], stressing a call to action for increased interventions for vitamin D, highlights a plateau and decrease in the incidence of type 1 DM that can be associated with the introduction of fortified milk. It should be noted that, in treatment studies, evidence was inconsistent as to the ability of vitamin D to be a supportive factor in reversing insulin resistance. Despite some positive studies, others concluded that therapeutic doses of vitamin D failed to improve insulin resistance alone [16].

Prevention

To prevent vitamin D deficiency and its associated health problems, recommendation guidelines are available for supplements at various ages [6]. Infants under the age of 12 months are to receive 400 IU each day. It is important to note that children receiving breast milk may need a vitamin D supplement, such as vitamin D drops. While breast milk may seem adequate, it has been found to lack the daily recommended amount of vitamin D. For children receiving infant formula, 32 ounces of formula is required to meet daily recommendations. Children between the ages of 12 and 24 months are to receive 600 IU per day [29,30]. Foods containing vitamin D, such as eggs and some fish, can be incorporated into a child's diet beginning at age two. The recommendations for fish are 2-3 servings per week and may include tilapia, tuna (light or canned; including skipjack), and salmon [10,29]. More importantly, it is recommended that children receive an adequate amount of sunlight to stimulate natural vitamin D synthesis in the body. Exposure to the sun may be moderate and can be obtained while children engage in outdoor physical activities. All caregivers must discuss diet and sun exposure with a provider to ensure children meet daily vitamin D recommendations [13]. The recommended dietary allowances for vitamin D, as per the National Institutes of Health, Office of Dietary Supplements, are presented in Table 6.

**Table 6:** RDAs for vitamin D *Adequate intake RDAs: recommended dietary allowances Source: [6]

Age	Male	Female	Pregnancy	Lactation
0-12 months*	10 mcg (400 IU)	10 mcg (400 IU)	-	-
1-13 years	15 mcg (600 IU)	15 mcg (600 IU)	-	-
14-18 years	15 mcg (600 IU)	15 mcg (600 IU)	15 mcg (600 IU)	15 mcg (600 IU)
19-50 years	15 mcg (600 IU)	15 mcg (600 IU)	15 mcg (600 IU)	15 mcg (600 IU)

Limitations

Although this systematic review confirmed the significant association between vitamin D deficiency, obesity, and insulin resistance, some limitations were found. Different studies included in this systematic review used different age groups, which resulted in a variable range between 0 months and 18 years. Most importantly, different articles had different definitions of vitamin D deficiency; ranges varied from 25 nmol/L to 50 nmol/L as a cutoff for the definition of deficiency. Due to the inconsistency in defining vitamin D deficiency, results may be affected in terms of whether or not the association between vitamin D deficiency, obesity, and insulin resistance is significant.

Additionally, little review was done into confounding factors. Lifestyle, diet, and sun exposure were not assessed at the time of the study and may affect the significance of the association. Finally, this systematic review is limited to childhood age and does not reflect on adults or maternal health.

Recommendations

Our findings yield some recommendations for consideration. Our review supports continued efforts in considering vitamin D status as an area of pediatric importance. Supplement recommendations and screening guidelines should continue to be at the forefront of pediatric position statements for nutritional health and intervention guidelines [26]. The strong association between vitamin D and obesity confirms continued reasoning for healthcare efforts to curb the well-known concerns of the pediatric obesity epidemic, along with the other comorbidities and burdens that come with obesity.

Additionally, given the apparent complex metabolic role vitamin D plays in the human body, recommendations would be to grow efforts in increasing screening as a consistent pattern in well-checks for the pediatric population. In concert with the standardization of screening, it is recommended that definitions and guidelines for defining vitamin D deficiency become more standardized as well.

Future Directions

At this current time, recommendations for screening for vitamin D include risk factors such as known absorptive disorders, known poor diets, and obesity. This supports that clinical attention toward the impact of finding deficiency and intervention has a trend in importance among pediatric practitioners. Possible future directions would be to continue efforts in developing research that focuses on the relationship between vitamin D and metabolic dysfunctions.

In light of the strong associative evidence of frank obesity and vitamin D, it would be important to continue efforts in interventions for obesity in both diet and an active lifestyle. Prevention of obesity alone would be argued to be one supportive factor in preventing vitamin D deficiency. Our studies did not specifically focus on activity as a confounding factor, and it would be an interesting route of study to determine causal and treatment effects of activity alone on vitamin D, separate from weight management. Further, vitamin D as a treatment to reverse conditions other than frank deficiency has weak evidence. Future directions could include determining what, if any, role vitamin D has as corrective therapy in other metabolic conditions.

In general, given this study's directive focus on the pediatric population, this yields time and potential for more specific long-term cohort studies. A deeper knowledge of the impact of vitamin D status across the lifespan could have an impact on developing recommendations for pediatric care for generations to come.

## Conclusions

This review yields a reasonable conclusion: our findings more support than reject the finding that vitamin D deficiency has a predictive association with health status in the pediatric population with obesity. This correlation has strong significance in both ethnicity and geographic location. However, broader correlative findings would support that this link goes beyond potentially cultural and geographical factors. Obesity alone does indicate to be an associated factor of vitamin D deficiency. It should therefore be encouraged to continue efforts to stress the importance of pediatric screening of vitamin D levels on evidence of obese individuals. Vitamin D, on the other hand, does not demonstrate a reciprocal effective intervention other than on corrective measures of serum levels.

Along with obesity, vitamin D further demonstrates relationships in more specific metabolic disorders such as impaired fasting glucose, lipids, and PTH, although with some conflicting findings. The mechanism behind these associations implicating vitamin D as a causal factor remains overall unclear. However, evidence certainly supports a close relationship in these metabolic concerns, to support the potential coexistence of these concerns and to consider broad testing and treatment accordingly.
